# Effect of Weight Regain on Body Composition and Metabolic Biomarkers After Sleeve Gastrectomy: a Cross-Sectional Study from a Hospital Database

**DOI:** 10.1007/s11695-022-06384-3

**Published:** 2022-12-03

**Authors:** Mohamed Hany, Hala M. Demerdash, Ahmed Zidan, Ann Samy Shafiq Agayaby, Bart Torensma

**Affiliations:** 1 Department of Surgery, Medical Research Institute, Alexandria University, 165 Horreya Avenue, Hadara, 21561 Alexandria Egypt; 2 Madina Women’s Hospital, (IFSO Centre of Excellence, European Chapter), Alexandria, Egypt; 3 Department of Clinical Pathology, Alexandria University, Alexandria, Egypt; 4 Leiden University Medical Center (LUMC), Leiden, The Netherlands

**Keywords:** Weight regain, Weight loss, Body composition, Metabolic biomarkers, Bariatric surgery, Sleeve gastrectomy

## Abstract

**Introduction:**

Weight regain (WR) is described in approximately 30% of patient’s post-bariatric surgery. It is related to the progression or recurrence of associated medical problems and decline in health-related quality of life. This study aimed to test the return of body composition and metabolic biomarkers to pre-operative levels when WR occurs.

**Methods:**

In this cross-sectional study conducted in 2021, patients were randomly selected from the hospital’s electronic databases between 2001 and 2020. Patient demographic data, comorbidities, body compositions, and metabolic biomarkers were collected. Three groups were defined: groups A (WR), B (weight loss), and C (control group; patients with obesity who had not yet undergone bariatric surgery).

**Results:**

A total of 88 patients were enrolled in this study and matched with the control group. The body mass index in group A was 43.8 ± 6.9 kg/m^2^; group B was 28.6 ± 4.2; group C was 43.9 ± 7.1. Body muscle mass, body fat mass, and visceral fat significantly differed between groups A and B (*p* < 0.001) but not between groups A and C (*p* = 0.8). There was a significant difference in leptin, ghrelin, postprandial glucagon-like peptide-1, insulin, and fibroblast growth factor-21 (but not retinol-binding protein-4) between groups A and B. Most metabolic biomarkers in group A returned to the pre-operative values as in group C.

**Conclusion:**

WR had a direct negative effect on body composition and metabolic biomarkers, whereby the values returned to pre-operative levels. Early detection of WR and possible additional therapy are necessary to prevent associated medical problems.

**Graphical Abstract:**

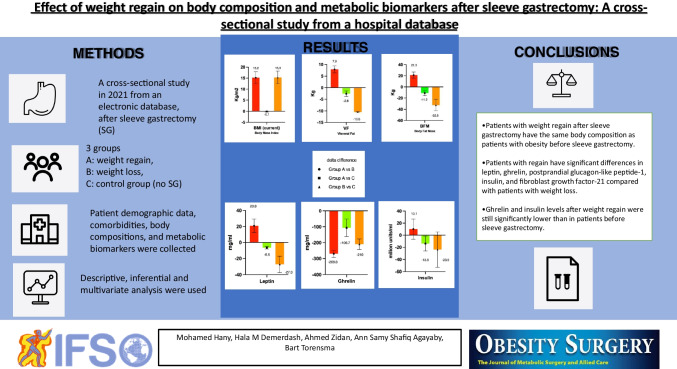

## Introduction

Bariatric surgery is an efficient procedure that can result in considerable sustained weight loss in patients with obesity, resolves associated medical problems with obesity, and improves quality of life [1]. Weight loss after bariatric surgery results in improvements in health, cellular processes, and metabolic biomarkers [2, 3]. Nevertheless, weight regain (WR) is described in approximately 30% of the patient’s post-bariatric surgery and is a challenge to many patients and may complicate long-term outcomes [4–8]. WR after a bariatric procedure is related to the progression or recurrence of associated medical problems and decline in health-related quality of life and satisfaction [4].

An important aspect of defining success after bariatric surgery is the long-term health effect.

Also, the effect of metabolic biomarkers is essential after weight loss since all metabolic biomarkers impact body composition by increasing and decreasing weight and the behavior of these hormones. Leptin is an adipocyte-derived hormone that mainly regulates energy storage within the body; thus, energy is generated by adipocytes in proportion to their triglyceride content [9]. Ghrelin, a stomach-derived hormone, promotes food intake and is involved in lipogenesis and insulin sensitivity [10].

Furthermore, glucagon-like peptide-1 (GLP-1) is released from the gut in response to food intake. It functions as a satiety signal and as an incretin hormone, acting on the pancreas to stimulate insulin release in a glucose-dependent manner [11, 12]. Retinol-binding protein-4 (RBP4) is synthesized in several tissues, primarily the liver and adipose tissues. RBP4 reportedly acts as an adipokine, linking adipocyte glucose metabolism with systemic insulin sensitivity [13]. Fibroblast growth factor-21 (FGF-21) is a novel metabolically active hormone that has been investigated, given its potential therapeutic role in obesity and metabolic recovery following bariatric surgery, particularly for diabetes. FGF-21 is secreted predominantly in the liver, with low secretion rates in the muscles, adipose tissues, and pancreas [14]. Leptin resistance is analogous to insulin resistance, and they often coexist in patients with obesity and change with changes in body composition and weight over time. The effect of weight loss after bariatric surgery on body composition and hormonal changes is known; however, the effect of WR after bariatric surgery remains uncertain.

Recently, a study by Luna et al.[15] tested WR after Roux-en-Y gastric bypass surgery on body composition and metabolic biomarkers. Nevertheless, the same concept is, to our knowledge, not yet performed on laparoscopic sleeve gastrectomy (LSG) patients, whereby the differences before and after weight loss and regain were tested. This study aimed to test the hypotheses that patients with WR have a relapse in body composition and metabolic biomarkers after LSG surgery.

## Methods

This was a cross-sectional study conducted in 2021 on body composition and metabolic biomarkers from a hospital electronic database selection between 2001 and 2020 at Madina Women’s Hospital, an IFSO-accredited center of excellence (European chapter) in Alexandria, Egypt. The study was conducted in accordance with the principles of the Declaration of Helsinki and approved by the ethical committee board.

### Patient Selection

Three groups were defined according to the Percentage Maximum Weight Loss (%MWL) criteria. This is the percentage of WR over the %MWL. This was calculated as (current weight − minimum weight) × 100/(pre-operative weight − minimum weight) [4, 16].

Therefore, patients were randomly selected, without further checking the patient information at that time, from the hospital’s electronic database who had undergone LSG surgery and met the %MWL criteria. Random selection was performed to overcome selection bias about the patient’s pre- or post-operative data. Written and oral informed consent was obtained before enrollment from all patients, and all the data were analyzed anonymously.

This group was invited for a cross-sectional assessment at the surgical outpatient clinic after.

- Group A: WR group; patients who presented with weight gain > 20% of the MWL.
- Group B: Weight loss group (WL group); patients who achieved %MWL ˂20%.
- Group C: Control group (Co group); pre-operative patients with obesity (matched by age and sex) who had not yet undergone bariatric surgery.

### Exclusion Criteria

Inability to sign an informed consent form.

### Data Collection

Data on patient demographics associated medical problems at the time of participation, body compositions, and metabolic biomarkers were collected.

### Methods of Determining Body Composition

The following parameters were collected for body composition: body mass index (BMI), body muscle mass (BMM), body muscles percentage (BM%), body fat mass (BFM), body fats percentage (BF%), and visceral fat (VF). Body composition was tested using electrical bioimpedance with an In-Body 120 device (South Korea) to assess BF%, BFM, FFM, skeletal muscle mass, and VF in the patients.

### LSG

First, all patients were pre-operatively screened and indicated for LSG surgery according to the International Federation for the Surgery of Obesity and Metabolic Disorders (IFSO) criteria. After that, the same team performed LSG whereby a dissection was initiated at 6 cm from the pylorus (antrum preserving) up to the gastroesophageal junction, followed by gastric transaction over a 40F bougie through sequential stapler firings.

### Laboratory Investigations for Metabolic Biomarkers

Peripheral blood samples were collected after overnight fasting; Serum samples were allowed to clot at room temperature for 30 min and subsequently centrifuged at 4000 rpm for 10 min at 4 °C. The serum was removed and stored at − 80 °C until further analysis.

### Blood Sampling Sequence

First, **fasting samples** were obtained to measure metabolic biomarkers, including leptin, ghrelin, insulin, RBP-4, and FGF-21. All the patients were subsequently provided with **a standard meal** (300 kcal) comprising 20 g of sliced bread, 15 g of butter, 100 g of Greek yogurt, 50 g of apple slice, and 6 g of walnuts, providing 20% protein, 35% fat, and 45% carbohydrates. The meal duration was approximately 20 min. Blood samples were collected immediately after and 120 min after ingesting the standard meal for **postprandial sampling** of GLP-1.

### Hormonal Measurements

All the measurements were analyzed according to standardized operating procedures. Lipid profile was determined using Hitachi 7180 Biochemistry Automatic Analyzer (Hitachi, Japan), and the hormones were assessed using ELISA (EIA-2935) (DRG International, Inc. Springfield NJ, USA) (Appendix).

### Statistical Analysis

Descriptive and inferential statistics were used for analyses. All the data were tested for normality using the Kolmogorov–Smirnov test, Q-Q plot, and Levene’s test. Categorical variables are expressed as numbers and percentages. Normally and non-normally distributed continuous variables are presented as means and standard deviations (SDs) and medians and interquartile ranges, respectively. When appropriate, categorical variables were tested using Pearson’s chi-square test or Fisher’s exact test. Normally distributed continuous data were tested with dependent samples using Student’s *t*-test for pre- and post-operative results. For skewed (non-parametric) data, the Wilcoxon signed-rank test was used. Predictors were evaluated using univariate and multivariate linear regression analyses.

All independent variables, including over 10 events with *p*-values < 0.1, were eligible for multivariate analysis, which was achieved through backward selection. The optimal prediction model was evaluated with a 2 log-likelihood test. *p*-values < 0.05 were considered significant. Statistical analyses were performed using R (version 4.0.4) packages.

### Sample Size Calculation

Sample size was calculated using R software version 4.1.3 and its “pwr” package. Several studies compared across groups the body composition and metabolic biomarkers using analysis of variance revealed an effect size of 0.25, 0.30, and 0.35, slightly close to the large effect size of 0.4 that is predicted. [17, 18] In this study, we conducted multiple linear regressions; therefore, a large effect size of 0.35 was used with a (1-β) power of 80% at *α* = 0.05, yielding a minimum sample size of 56.

## Results

### Baseline Characteristics

Eighty-eight patients were randomly enrolled in this study (30 patients each in groups A and B matched with 28 patients in group C). All patients in groups A and B underwent LSG. Mean (± SD) age was 43.6 ± 10.5, 38.3 ± 9.7, and 36.5 ± 12.2 years, in groups A, B, and C, respectively, and all the groups had more women (A = 66.7%, B = 83.3%, and C = 67.9%). Associated medical problems in groups A and C were more significant than those in group B [dyslipidemia (20% and 17.9% vs. 0%), asthma (10% and 17.9% vs. 0%), and sleep apnea (20% and 21.4% vs. 0%); *p* = 0.02, 0.04, and 0.02, respectively].

The follow-up period in group A, which had more than 4 years (range, 2001–2018), was 93.4%, 3.3% of the patients had three, and 3.3% had 2 years of follow-up (total 100%). In group B, all the patients were followed up for 2 or 3 years after LSG (Table 1).

**Table 1:** Baseline characteristics

Characteristics	Group A *N* = 30	Group B *N* = 30	Group C *N* = 28	*p*-value
Age years (mean ± SD)	43.6 ± 10.5	38.3 ± 9.7	36.5 ± 12.2	**0.04**
Sex (female)	20 (66.7%)	25 (83.3%)	19 (67.9%)	0.28
BMI (kg/m^2^) (mean ± SD)				
BMI (before LSG)	50.7 ± 10.8	47.1 ± 7.9	-	0.128
BMI (nadir)	35.06 ± 7.68	-	-	-
BMI (current)	43.8 ± 6.9	28.6 ± 4.2	43.9 ± 7.1	**A vs B 0.001** **A vs C 0.80** **B vs C 0.001**
Comorbidities *n* (%)				
Hypertension	6 (20%)	3 (10%)	8 (28.6%)	0.20
Diabetes mellitus	3 (10%)	3 (10%)	4 (14.3%)	0.84
Dyslipidemia	6 (20%)	0 (0%)	5 (17.9%)	**0.02**
Ischemic heart disease	0 (0%)	0 (0%)	1 (3.6%)	0.32
Asthma	3 (10%)	0 (0%)	5 (17.9%)	**0.04**
Sleep apnea	6 (20%)	0 (0%)	6 (21.4%)	**0.02**
Hypothyroidism	3 (10%)	0 (0%)	2 (7.1%)	0.27
History of DVT	0 (0%)	1 (3.3%)	0 (0%)	1
Rheumatoid arthritis	1 (3.3%)	0 (0%)	1 (3.6%)	0.76

**Group A**: weight regain group (WR group). **Group B**: weight loss group (WL group). **Group C**: control group (Co group); pre-operative patients with obesity

*BMI*, body mass index; *SD*, standard deviation; *DVT*, deep vein thrombosis; *LSG*, laparoscopic sleeve gastrectomy

### Multiple Regression Analysis

After univariate regression analysis, multivariate regression analysis was conducted on adjusted variables, including age, sex, and associated medical problems, with a mean pairwise difference in body composition and metabolic biomarkers. In addition, the differences between groups A (weight regain) and B (weight loss), groups A and C (control group), and groups B and C were analyzed.

### BMI

#### BMI Before LSG

The mean BMI (± SD) was 50.7 ± 10.8 kg/m^2^ in group A and 47.1 ± 7.9 kg/m^2^ in group B; the difference in BMI between groups A and B was not significant (*p* = 0.128) (Table 1).

#### Nadir’s BMI After LSG

Nadir’s BMI was 35.06 ± 7.68 kg/m^2^ in group A (Table 1).

#### Current BMI After LSG in Groups A and B Compared with that in the Control Group

The post-LSG BMI was 43.8 ± 6.9 kg/m^2^ in group A (WR group), 28.6 ± 4.2 kg/m^2^ in group B (weight loss group), and 43.9 ± 7.1 kg/m^2^ in group C (control group).

**Fig. 1:**
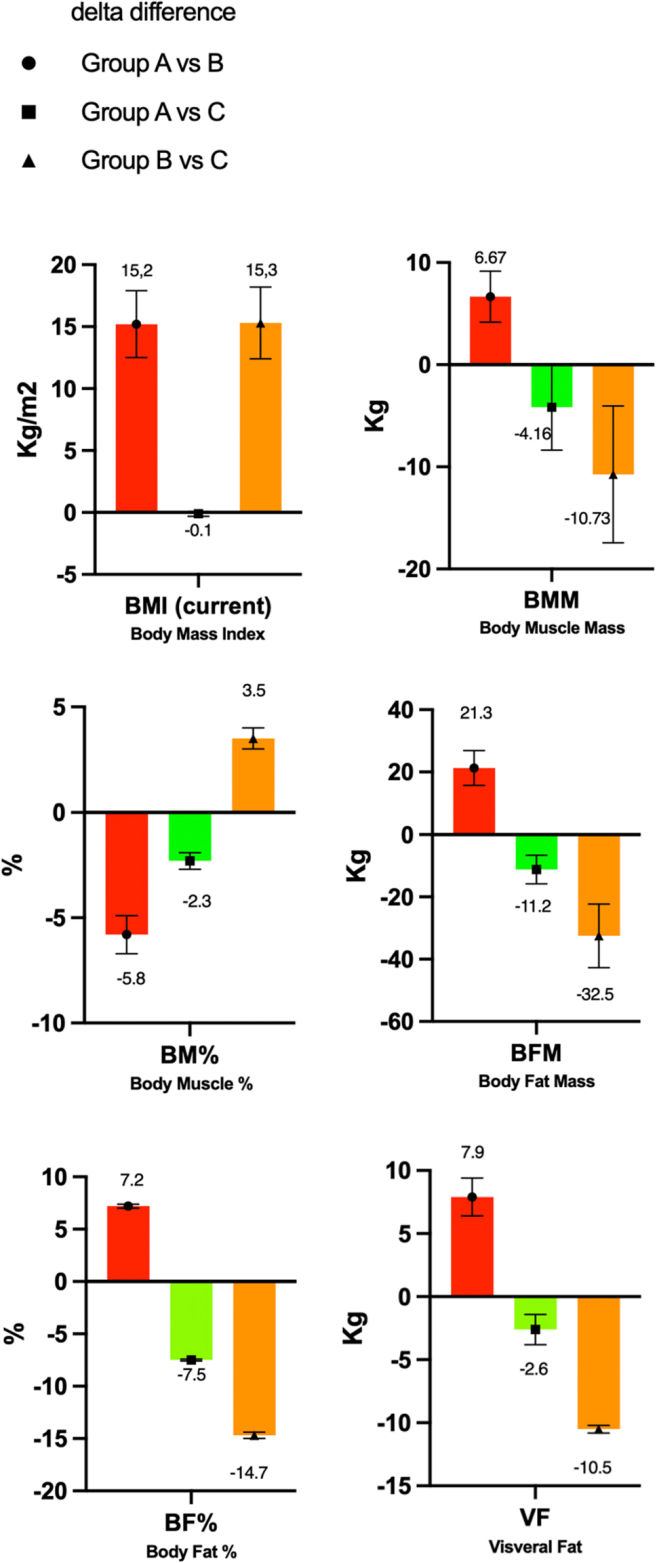
Body compositions

Post-LSG BMI significantly differed between groups A and B (*p* < 0.001) and B and C (*p* < 0.001) but not between groups A and C (*p* = 0.80) (Fig. 1).

#### Percentage Maximum Weight Loss (%MWL)

The median (IQR) percentage of WR relative to the %MWL was 60.2% (67.5%) in group A.

### Body Composition

#### Body Muscle Mass (BMM)

BMM was 31.9 ± 6.1 kg, 25.23 ± 3.6 kg, and 36.06 ± 10.3 kg in groups A, B, and C, respectively.

BMM significantly differed between groups A and B (*p* < 0.002) and B and C (*p* < 0.001), but not between groups A and C (*p* = 0.08) (Table 2; Fig. 1).

**Table 2:** Body composition and serum levels of gut hormones. Adjusted^a^ mean pairwise differences in body composition and gut hormones

Characteristics	Group A *N* = 30	Group B *N* = 30	Group C *N* = 28	*p*-value A vs B	*p*-value A vs C	*p*-value B vs C
Body composition						
BMM (kg) mean ± SD	31.9 ± 6.1	25.23 ± 3.6	36.06 ± 10.3	** < 0.001**	0.08	** < 0.001**
BM % mean ± SD	26.7 ± 2.9	32.5 ± 3.8	29.0 ± 3.3	** < 0.001**	0.08	**0.006**
BFM (kg) mean ± SD	44.9 ± 10.8	23.6 ± 5.2	56.1 ± 15.4	** < 0.001**	** < 0.001**	** < 0.001**
BF % mean ± SD	37.4 ± 4.4	30.2 ± 4.2	44.9 ± 4.5	** < 0.001**	** < 0.001**	** < 0.001**
VF (kg) mean ± SD	16.5 ± 2.7	8.6 ± 1.2	19.1 ± 1.5	** < 0.001**	< 0.8	** < 0.001**
Metabolic biomarkers						
Leptin (mg/ml) Mean ± SD	40.6 ± 13.3	19.8 ± 4.9	47.1 ± 15.1	** < 0.001**	0.06	** < 0.001**
Ghrelin (mg/ml) mean ± SD	372.7 ± 46.8	269.1 ± 69.1	479.1 ± 102.1	** < 0.001**	** < 0.001**	** < 0.001**
Postprandial GLP-1 (mg/ml) median (IQR)	3.67 (0.55)	4.1 (9.9)	4.7 (0.7)	**0.002**	0.27	0.33
Insulin (million units/ml) median (IQR)	17.9 (21.35)	7.8 (4.62)	31.7 (33.7)	** < 0.001**	** < 0.001**	** < 0.001**
RBP-4 (mg/ml) median (IQR)	34.8 (40.2)	41.3 (46.9)	34.8 (26.9)	0.08	0.72	0.17
FGF-21 (pg/ml) median (IQR)	281 (297)	105 (71)	268 (280)	** < 0.001**	0.051	** < 0.001**

**Group A**: weight regain group (WR group). **Group B**: weight loss group (WL group). **Group C**: control group (Co group); pre-operative patients with obesity. *BMI*, body mass index; *BMM*, body muscle mass; *BM %*, body muscles percentage; *BFM*, body fat mass; BF %, body fats percentage; *VF*, visceral fat; *SD*, standard deviation

^a^Adjusted for age, sex, and comorbidities

#### Body Muscles Percentage (BM%)

BM% was 26.7 ± 2.9, 32.5 ± 3.8, and 29.0 ± 3.3 in groups A, B, and C, respectively.

**Fig. 2:**
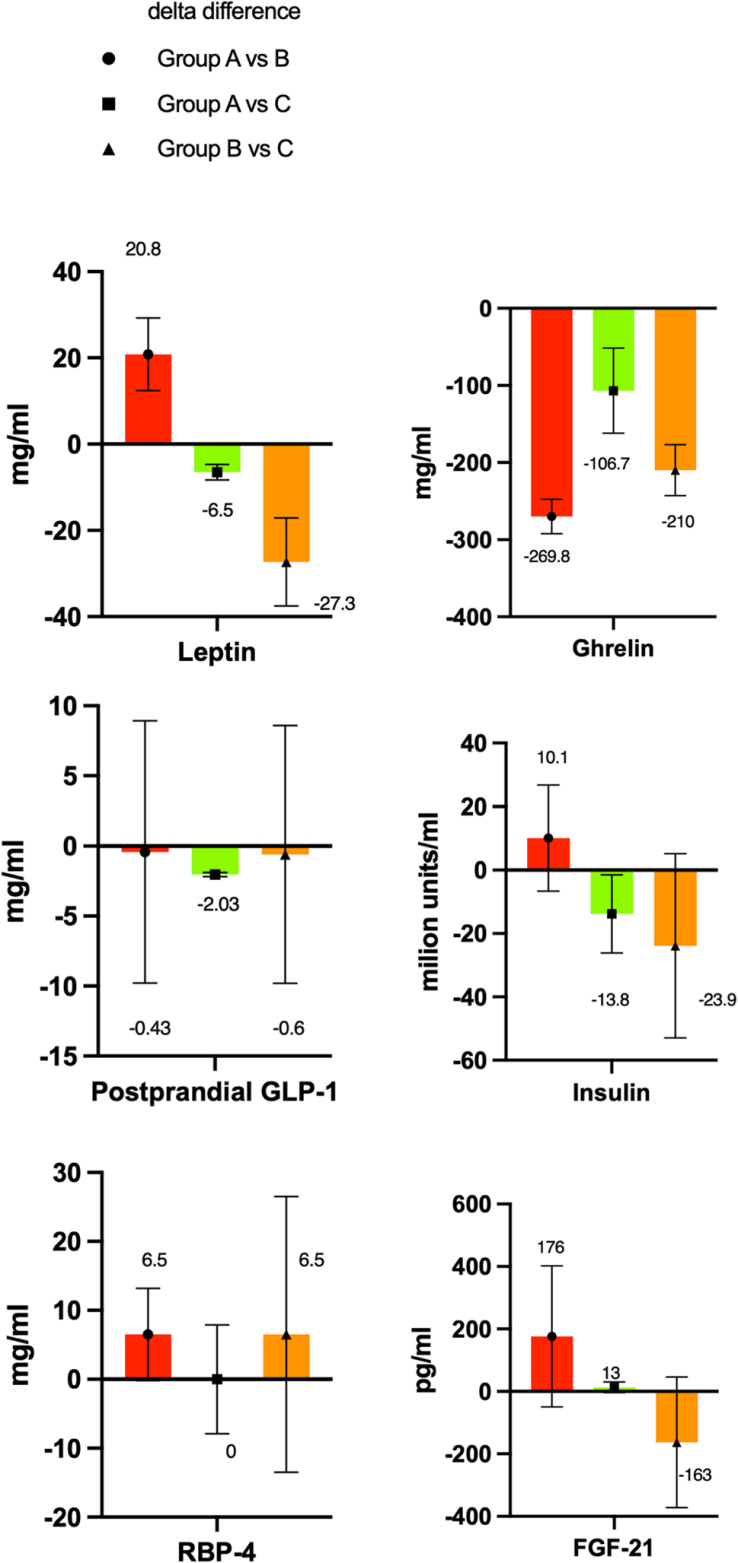
Metabolic biomarkers

It significantly differed between groups A and B (*p* < 0.001) and B and C (*p* = 0.006) but not between groups A and C (*p* = 0.08) (Table 2; Fig. 2).

#### Body Fat Mass (BFM)

BFM was 44.9 ± 10.8 kg, 23.6 ± 5.2 kg, and 56.1 ± 15.4 kg in groups A, B, and C, respectively. It significantly differed between groups A and B, A and C, and B and C (all *p* < 0.001) (Table 2; Fig. 1).

#### Body Fats Percentage (BF%)

BF% was 37.4 ± 4.4, 30.2 ± 4.2, and 44.9 ± 4.5 in groups A, B, and C, respectively.

It significantly differed between groups A and B, A and C, and B and C (all *p* < 0.001) (Table 2; Fig. 1).

#### Visceral Fat (VF)

VF was 16.5 ± 2.7 kg, 8.6 ± 1.2 kg, and 19.1 ± 1.5 kg in groups A, B, and C, respectively.

It significantly differed between groups A and B and groups B and C (both *p* < 0.001), but not between groups A and C (*p* = 0.8) (Table 2; Fig. 1).

### Metabolic Biomarkers

#### Groups A and B

The differences in leptin (40.6 ± 13.3 vs. 19.8 ± 4.9), ghrelin (372.7 ± 46.8 vs. 269.1 ± 69.1), postprandial GLP-1 [median (IQR), 3.67 (0.55) vs. 4.1 (9.9)], insulin [median (IQR), 17.9 (21.35) vs. 7.8 (4.62)], and FGF-21 [median (IQR), 281 (297) vs. 105 (71)] levels between groups A (WR) and B (weight loss) were significant (*p* < 0.001, < 0.001, = 0.04, and 0.004, respectively).

However, the difference in RBP-4 levels was not significant [median (IQR): 34.8 (40.2) vs. 41.3 (46.9); *p* = 0.08]; Table 2; Fig. 2].

#### Groups A and C

The differences in ghrelin (372.7 ± 46.8 vs. 479.1 ± 102.1) and insulin [median (IQR), 17.9 (21.35) vs 31.7(33.7)] levels between groups A and C (control) were significant (both *p* < 0.001) (Table 2; Fig. 2).

However, the difference in leptin (40.6 ± 13.3 vs. 47.1 ± 15.1), postprandial GLP-1 [median (IQR), 3.67 (0.55) vs. 4.7 (0.7)], RBP-4 [median (IQR), 34.8 (40.2) vs. 34.8 (26.9)], and FGF-21 [281 (297) vs. 268 (280)] levels was not significant (*p* = 0.06, 0.27, 0.72, and 0.051, respectively) (Table 2; Fig. 2).

#### Groups B and C

The differences in leptin (19.8 ± 4.9 vs. 47.1 ± 15.1), ghrelin (269.1 ± 69.1 vs. 479.1 ± 102.1), insulin [median (IQR), 7.8 (4.62) vs. 31.7 (33.7)], and FGF-21 [median (IQR), 105 (71) vs. 268 (280)] levels between groups B and C were significant (all *p* < 0.001).

In contrast, differences in RBP-4 [median (IQR); 41.3 (46.9) vs. 34.8 (26.9)] and postprandial GLP-1 [median (IQR); 4.1 (9.9) vs. 4.7 (0.7)] levels were not significant (*p* = 0.17 and 0.33, respectively) (Table 2; Fig. 2).

## Discussion

This study showed that after nadir’s BMI in group A, the BMI increased significantly again compared to group B (*p* =  < 0.001). And that the BMI compared with the control group C was not considerably different anymore and was almost back to the same baseline as before LSG surgery. Furthermore, all the body compositions (body fat, body muscle, and VF) did not significantly differ between the WR and control groups (A and C). However, they differed between both groups and group B (the weight loss group).

### WR and Body Compositions

BMI, as a predictor of the risk for metabolic complications, remains insufficient [19]. In patients with chronic diabetes, there is a significant reduction in post-operative disease remission [20, 21].

Associated medical problems are linked with obesity, and high VF can be identified in a higher percentage of people with hypertension, hyperglycemia, dyslipidemia, and other cardiovascular risk factors [22, 23]. It is vital to understand the effect of body composition on patients with obesity, pre- and post-bariatric surgery. Abdominal obesity is a basis for metabolic syndrome, which is linked to insulin resistance, hypertension, hyperlipidemia [24], and increased BMI-independent harm [25, 26]. Body fat distribution is highly associated with metabolic disorders; this was observed in a study by Ambrosi et al., who presented that irrespective of BMI or weight loss, the increase in body fat percentage after Roux-en-Y gastric bypass (RYGB) is associated with cardiometabolic risk factors [27]. Changes in body composition and weight loss were observed in two studies after LSG operation, which showed all decreased levels after the weight loss [17, 18]. Luna et al. [15] observed the same effect in RYGB patients; our study confirms this. Changes in body composition and weight loss were also observed in a study by Otto in 2016, whereby LSG and RYGB did not differ in terms of body composition and weight loss 1 year after surgery [28]. Three studies [17, 18, 28] tested this in one group of patients who lost weight and not in a group with WR. Luna et al. also tested that after 60 months of WR following RYGB, the body composition almost completely returned to that of the pre-operative obesity [15]. We also found the same results post-LSG, with a significant increase in the body composition and VF in group A compared with group B after WR. Furthermore, there were no significant differences between group A and the control group C. Several studies observed that 70–80% of weight loss occurs due to reduced fat mass also to decreased adipocyte size [29, 30]. The proportion of VF changes the most due to the high lipid levels. Decreased VF is linked with the metabolic benefits of surgery [30–32], which are reversed with WR. This implies that after a period of WR (group A), the body composition returns to the pre-operative level, along with all the consequences and associated medical problems [23, 32–36].

### WR and Metabolic Biomarkers

This study also revealed the return of metabolic biomarkers after WR. Several studies have revealed the effect and benefits of weight loss surgery on metabolic biomarkers like gut hormones, insulin levels, and other adiposity signals [14, 37–44]. While hormones exert beneficial effects on metabolic biomarkers after weight loss, WR was found to exert negative effects in this study.

### Leptin

Leptin decreases food intake while increasing energy expenditure and initiating weight loss, and higher body fat results in more leptin being produced. This causes leptin resistance, affecting the satiety signal and resulting in the feeling of not being full or satiated. This study revealed that as body fat increases, leptin levels rise. Additionally, there is cellular leptin resistance through various conduits, such as genetic alterations and variations in peripheral tissues that favor adiposity independently of food intake changes [45].

### Ghrelin

Bariatric surgery instantly affects ghrelin levels, reducing them after weight loss due to reduced ghrelin-producing cells after the stomach is removed during LSG or disconnected during RYGB [46]. A systematic review in 2020 studied the effect of ghrelin and observed that all 25 studies (*n* = 604 patients) that reported fasting ghrelin levels demonstrated a decrease after LSG; this is consistent with our study’s results for group B. Nevertheless, they concluded that whether the decrease in ghrelin levels is sustained remains unknown. Luna [15] and the present study confirm that after weight loss surgery, ghrelin levels increase and negatively affect food intake when weight is regained.

### Postprandial GLP-1

Two systematic reviews and meta-analyses assessed the effect of postprandial GLP-1 after LSG and RYGB and revealed that post-operative GLP-1 was increased [46, 47], suggesting a similar hormonal mechanism of action. This finding may explain the significant improvement in glycemic control observed after RYGB and LSG. This study also observed an increase in fasting GLP-1 levels in group B; however, the opposite was observed in groups A and C (the levels decreased again when weight increased, negatively affecting food intake).

### Insulin

Obesity is a major risk factor for insulin resistance (IR), hyperinsulinemia, and type 2 diabetes mellitus (T2DM) [48]. LSG is a surgical intervention that, in addition to causing significant weight loss, is associated with early and high T2DM resolution rate [49] and the independent of the loss of fat mass, suggesting that it is more than a simple restrictive procedure [50]. The precise mechanisms involved in early blood glucose control after LSG remain unclear [40]. Notably, leptin and insulin act in the same key hypothalamic areas to decrease food intake and increase energy expenditure, thereby regulating long-term energy balance [51]. The effect of increased insulin levels (even if the normal insulin level is below the threshold of < 18–20 million units/ml) observed after LSG in group A (WR group) confirms their impact on changes in body composition (which is also observed in group B) when weight loss and normal body compositions are still present, positively affecting the levels of insulin. Patients in group C, who had not undergone surgery, had the highest insulin levels. This is possibly explained by the time between weight loss and the weight regain after LSG, which is still in progress, or suggesting that LSG might have a persisting effect post-operatively. However, more research on this is necessary.

### RBP-4

A study analyzed RBP-4 in patients with obesity after weight reduction followed by an improvement in obesity-related medical problems and found that the systemic concentration of RBP4 was lower than that reported in some studies [52–54]. The present study could not confirm whether WR caused an increase in RBP-4 levels since the differences between groups A and B were not significant; group A had lower RBP-4 values than group B [34.8 (40.2) vs. 41.3(46.9)], and the reasons for this remain unclear.

### FGF-21

A study observed that FGF21 levels were higher in patients with obesity than in patients with normal weight. A month after bariatric surgery, patients with obesity exhibited a significant increase in FGF21 levels [55]. A systematic review confirmed that FGF-21 increased after RYGB and observed that it decreased ≥ 1 year post-operatively [14]. The present study observed that prior to bariatric surgery, patients in group C had significantly higher FGF-21 levels compared to those in group B and that group A had increased FGF-21 levels. We cannot conclude on the events of the immediate post-operative period; however, after > 1 year, decreased FGF-21 levels were observed in patients without obesity compared with those with obesity, which is consistent with previous studies. The reason for the rise in FGF21 values shortly after bariatric surgery remains unclear; however, WR negatively impacts FGF-21 and body composition. One of the first studies that investigated the influence of gut hormones on WR after RYGB was conducted by Santo in 2016 [56], and a difference in the secretion of gut hormones were observed between patients with WR and those with weight loss after RYGB. This study had a small sample size and no control group to compare WR with [56]. To our knowledge, Luna [15] was the first to conduct thorough research involving a control group and long-term follow-up for patients that underwent RYGB. Together with our study results, we can conclude that WR negatively affects metabolic biomarkers and body composition. The timing for detecting WR is essential to prevent the recurrence of associated medical problems, psychological factors, and other health-related risks due to increased body composition and biomarkers. In a systematic review of 15 studies on WR after bariatric surgery by King et al. [4], the WR assessment timing range was 3–10 years post-operatively. This is consistent with the follow-up of the WR group in the present study. So, early detection of insufficient weight loss or possible WR is necessary within the first 3 years when WR is visible. A study observed that early post-operative weight loss can be used to identify patients whose predicted weight loss trajectories are not optimal [57]. A study in 2021 on early post-operative weight loss as a predictive variable for the 5-year outcome found a positive association between weight loss at 3 and 12 months and that at 60 months; non-responders in the first 3 months also had poor outcomes at 60 months [58]. Attention must be paid to the post-operative weight-loss period and patients with WR must be detected; moreover, additional training and assessments [59] or medication therapy may help. Furthermore, as a last resort against WR, revisional surgery using another procedure or a band procedure could be considered [60, 61].

### Limitations

This was a cross-sectional study done on a random sample from a database, which could have had a positive effect on selection bias since the selection was not dependent on a person. Nevertheless, this study design prevented long-term follow-up because a before and after measurement within a patient was lacking. So prognostic cohort studies are required to understand the trend in body composition changes and metabolic biomarkers within and between groups of patients better. Furthermore, early detection of WR or long-term cohort follow-up could provide more insights into the WR challenge. Also, a larger cohort will provide better insights into the possible confounding factors and bias, which were challenging to test in this study. Consequently, correlation and prediction modeling between and within body composition and metabolic biomarkers were impossible, and the extended statistical testing for this study was low.

## Conclusion

WR negatively affects body composition and metabolic biomarkers, as all the results returned to the pre-operative values. Therefore, early detection of WR and additional therapy are necessary to prevent obesity-associated medical problems.

## Appendix

### Hormonal Measurements

Serum glucose, total blood cholesterol, high density lipoprotein (HDL) cholesterol, and triglycerides were measured enzymatically on a Hitachi 7180 Biochemistry Automatic Analyzer (Hitachi, Japan), while low-density lipoprotein (LDL) cholesterol was afterwards calculated using the Friedewald’s formula. Fasting insulin levels were measured using ELISA (EIA-2935) [DRG International, Inc. Springfield NJ, USA].

Serum ghrelin was measured using ELISA Kit (Cloud- Clone Corp; cat no: E-01720hu) [W. Fernhurst Dr., Unit 2201, Katy, TX 77,494, USA]). Serum leptin was evaluated by ELISA Kit (Cloud- Clone Corp; cat no: E-00916hu) (TX 77,494, USA). Leptin/ghrelin ratio was calculated in arbitrary units as leptin in ng/ml multiplied by 103 and divided by ghrelin in pg/ml.

Glucagon peptide 1 quantified using ELISA Kit (Cloud- Clone Corp; Cat no: E-00658hu) (TX 77,494, USA). Human Peptide YY measured by ELISA Kit (Cloud- Clone Corp; Cat no: E-01191hu) (TX 77,494, USA). Serum retinol binding protein 4 determined by ELISA kit (Cloud- Clone Corp; cat no: SEA929Hu) (TX 77,494, USA).

Homeostasis model assessment of insulin resistance (HOMA-IR) was used to evaluate insulin resistance (fasting serum insulin (μIU/ml) × fasting plasma glucose (mmol/L)/22.5).
